# High Precision Digitization of Paper-Based ECG Records: A Step Toward Machine Learning

**DOI:** 10.1109/JTEHM.2019.2949784

**Published:** 2019-11-07

**Authors:** Mohammed Baydoun, Lise Safatly, Ossama K. Abou Hassan, Hassan Ghaziri, Ali El Hajj, Hussain Isma’eel

**Affiliations:** 1 Beirut Research and Innovation Center Beirut 2052 6703 Lebanon; 2 Electrical and Computer Engineering DepartmentAmerican University of Beirut 11238 Beirut Lebanon; 3 Internal Medicine DepartmentAmerican University of Beirut 11238 Beirut Lebanon

**Keywords:** Electrocardiogram, digitization, Matlab tool, image processing

## Abstract

Introduction: The electrocardiogram (ECG) plays an important role in the diagnosis of heart diseases. However, most patterns of diseases are based on old datasets and stepwise algorithms that provide limited accuracy. Improving diagnostic accuracy of the ECG can be done by applying machine learning algorithms. This requires taking existing scanned or printed ECGs of old cohorts and transforming the ECG signal to the raw digital (time (milliseconds), voltage (millivolts)) form. Objectives: We present a MATLAB-based tool and algorithm that converts a printed or scanned format of the ECG into a digitized ECG signal. Methods: 30 ECG scanned curves are utilized in our study. An image processing method is first implemented for detecting the ECG regions of interest and extracting the ECG signals. It is followed by serial steps that digitize and validate the results. Results: The validation demonstrates very high correlation values of several standard ECG parameters: PR interval 0.984 +/−0.021 (p-value < 0.001), QRS interval 1+/− SD (p-value < 0.001), QT interval 0.981 +/− 0.023 p-value < 0.001, and RR interval 1 +/− 0.001 p-value < 0.001. Conclusion: Digitized ECG signals from existing paper or scanned ECGs can be obtained with more than 95% of precision. This makes it possible to utilize historic ECG signals in machine learning algorithms to identify patterns of heart diseases and aid in the diagnostic and prognostic evaluation of patients with cardiovascular disease.

## Graphical abstract

We present a MATLAB-based tool and algorithm that converts a printed or scanned ECG into a digitized ECG signal. Validation on 30 ECG scanned curves more than 95% precision. This makes it possible to utilize historic ECG signals in machine learning algorithms to identify patterns of heart diseases and aid in the diagnosis and evaluation of patients with cardiovascular disease.

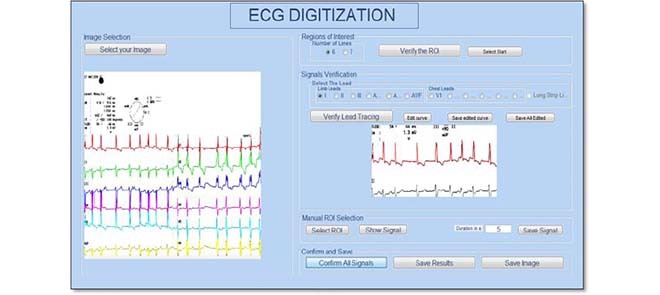

## I. Introduction

The electrocardiography (ECG) provides physicians with temporal and anatomic data about the heart reflected by the electric vector. In the right hands, it remains an essential tool in cardiac diagnosis since its introduction in 1902. Most patterns of cardiac diseases are based on old datasets and stepwise algorithms through interpretation of ECG findings. As such, the subjective assessment by the interpreter is the main drawback that is continuously addressed [1]. The transition of ECG charting from paper-based form to electronic signals allowed for instantaneous incorporation of patient specific data which are friendlier to analyze [2]. Current digitized waveforms pick up on objective parameters such as PR, QRS, QT intervals, ST elevation or depression and others, as well as advanced readings such as the morphology of the T-wave or the spatial QRS-T angle and many others previously overlooked.

The advances in machine learning in a wide spectrum of clinical applications have had major impact in fields related to signals such as ECGs [3], [4]. The big-data acquisition from signal datasets make them well suited for machine learning approaches. The use of machine learning tools on paper-based ECG cases is hindered by a translational stage that requires a stepwise identification of the ECG pattern. The first step is thus to achieve a fully reliable and reproducible digitized waveform from paper-based ECGs. The digitization process is normally performed by implementing adaptive and iterative digital image processing algorithms that transform the printed image into a set of time series digitized signals. Several methods are suggested in the literature to achieve acceptable digitization accuracy. Ravichandran et al used a Matlab-based tool that is based on the contour detection of the ECG signal after removing the background grid through thresholding [5]. Their work achieves batch processing of multiple ECGs by utilizing Optical Character Recognition (OCR) to extract the medical information of the patient that is available in the scanned record.

The proposed work is compared with previous works and the differences are highlighted. In particular, the work in [6] can be considered relatively simple especially that it utilized colored images. Other works such as [7] concentrated also on colored images. In addition, the work in [8] achieved an accuracy of 95% heart rate accuracy in comparison with a 100% heart rate accuracy in this work. This work compares also with the two most relevant works [9] and [5].

Therefore, in comparison with previous works, the proposed research provides several contributions. The first main contribution is the automated detection of each of the leads which was not performed in any previous work, but rather slightly addressed in [9]. Also, the work is applicable to ECGs with different resolutions and is not restricted to a specific dpi such as 300 and 600 dpi as in [5] or [9]. In addition, this work provides high accuracy when compared with the available approaches as can be seen later in the text.

This paper presents an improved method that provides high performance in comparison with prior techniques by dividing the digitized translation process into two main tasks: the detection of the regions of interest followed by the extraction of the path of each signal inside the detected boundaries. In addition, an accurate Matlab-based [10] menu-driven digitization tool for ECG paper-based records is presented. This tool is tailored for cardiologists and experts in the cardiovascular medicine field.

## II. Methods

### A. Stage I. Preprocessing

**FIGURE 1.**
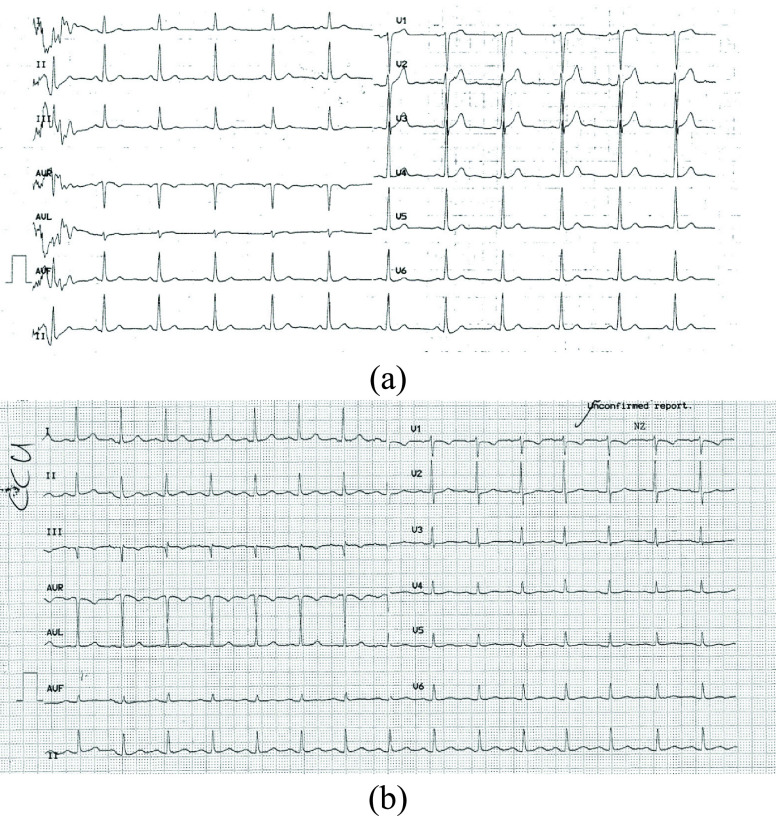
(a) Example of a printed ECG record, (b) example of a printed ECG with a graphical grid.

We mainly used paper-based ECGs retrieved from the electronic medical records of patients admitted to the American University of Beirut Medical Center. Data collection was performed in accordance with the protocols approved by the Institutional Review Board at AUBMC. Two printed ECG images are illustrated in Figures 1(a) and 1(b) showing the noise and undesired characters that can complicate the extraction process. The image shown in Figure 1(a) had a resolution of $1638\times 1147$ while that of Figure 1(b) had a $1725\times 1268$ resolution noting that both images have a png format which is the case for all ECG records obtained from AUBMC. We relied on these images which exceeded a 100 records to develop the proposed approach. We note that these images required creating a preprocessing step to filter noise and enhance the image. These records were not utilized in assessing the performance of the work as noted when discussing the results.

Other tools were also added to the preprocessing technique. Usually, the ECG output image is presented in 12 or 13 lead signals depending on the user preference and the ECG machine interface. These contain 12 lead signals corresponding to the electrode outputs that are placed on the patient’s limbs, on the surface of the chest and, if present, the addition of a long strip of lead II. The preprocessing step allowed the creation of 12 or 13 time series digitized signals; this was performed by implementing an adaptive and iterative set of digital image processing algorithms. The proposed tool offered the user an interface to enter the number of leads and to select the desired $13^{\mathrm {th}}$ lead.

### B. Stage II. Detecting Regions of Interest

**FIGURE 2.**
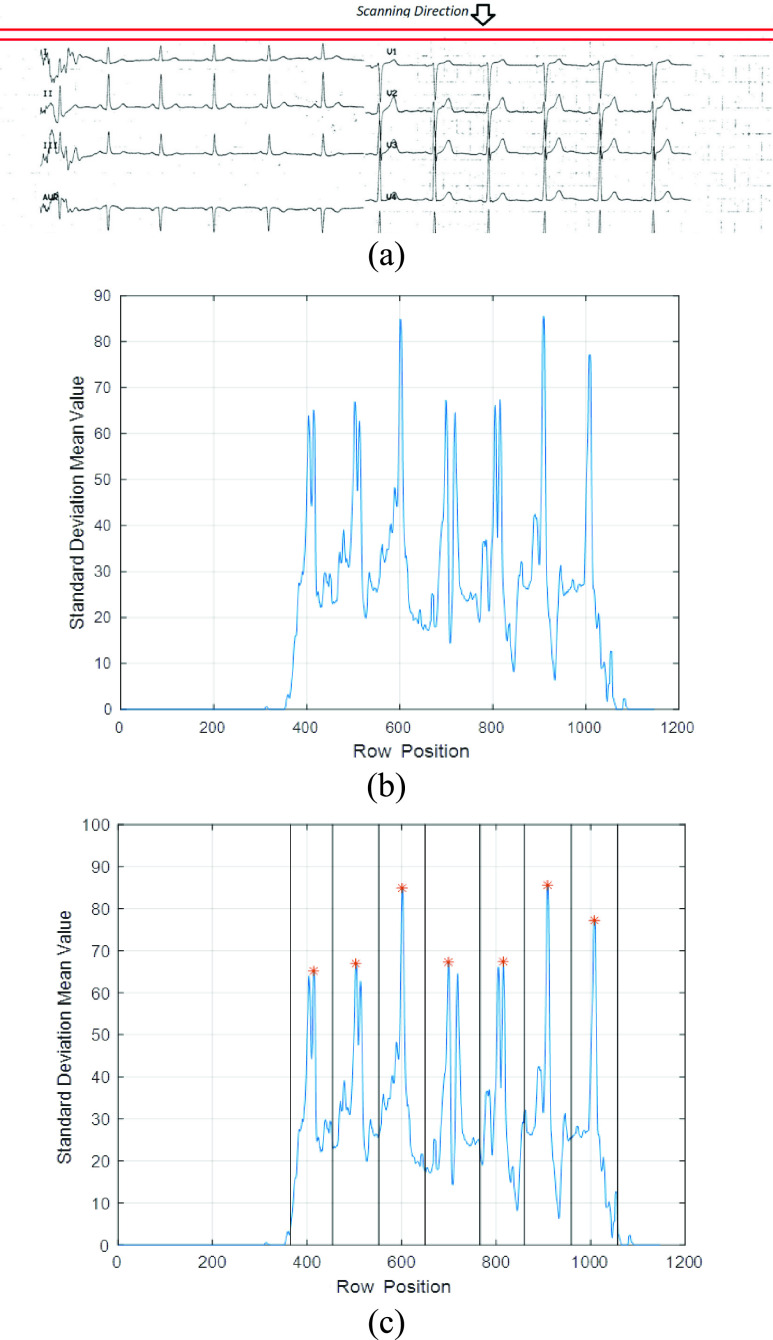
(a) Example of Scanning the ECG image along the vertical axis, (b) example of the obtained standard deviation vector for an input ECG image, (c) example of the obtained row based standard deviation vector for an input ECG image with the relevant peaks and regions highlighted.

In this stage, a bounding rectangle was determined for each signal showing its related region on the ECG image. The bounding rectangles were located by applying a mask composed of five rows over the whole image along the vertical direction as shown in Figure 2(a). This mask computed the standard deviation of each mask area that was composed of the main row and its adjacent ones. Clearly, an area with a mix of black and white pixels would have a high standard deviation. This would lead to a vector value that corresponds to the mean standard deviation of the mask. The obtained vector for the input image shown in Figure 1(a) is shown in Figure 2(b). The size of the vector is equal to the number of the rows of the input image.

The standard deviation vector curve in Figure 2(b) identified seven distinguishable areas which correspond to the 7 ECG lines. Using a basic peak detection algorithm, where the peak value always exceeds half of the maximum value (85 in this case) and with a minimum peak to peak distance that exceeds 5% of the length of the computed vector, we detected the centers of the different bounding boxes as shown in Figure 2(c). After detecting the peaks, we estimated the region of each signal by considering a rectangle around the peak which allowed to determine the horizontal boundaries for each signal.

**FIGURE 3.**
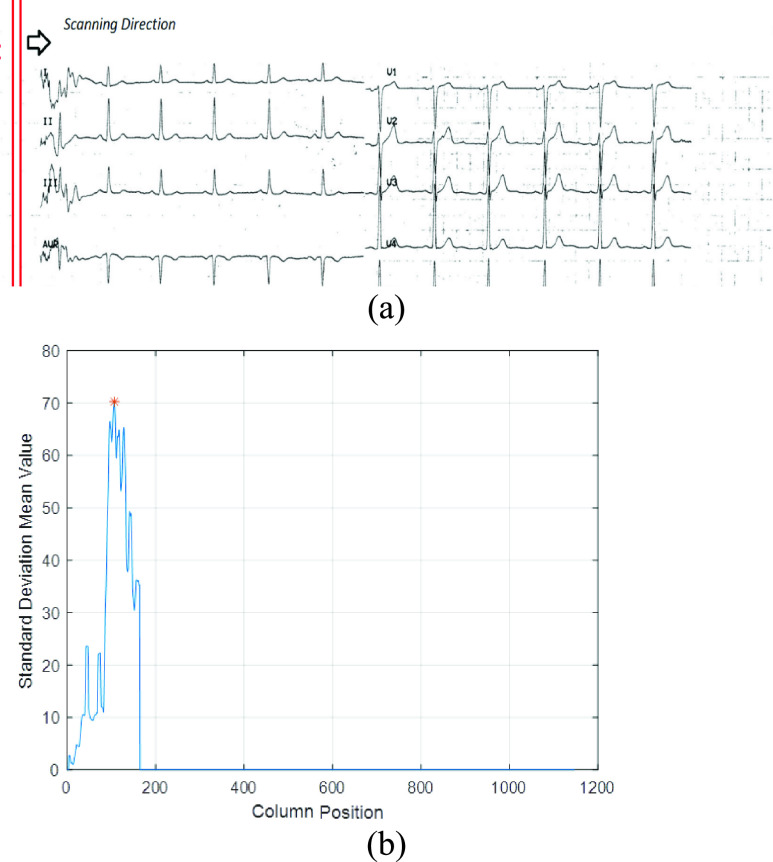
(a) Example of Scanning the ECG image along the horizontal axis, (b) example of the obtained column based standard deviation vector for an input ECG image with the relevant peak.

The same approach was used to determine the vertical boundaries. The mask composed of five columns was passed to locate the starting position as shown in Figure 3(a). In this case, the mask was only passed over the first ten percent of the columns (10%) while computing the standard deviation of the current mask area. The output vector allowed for determining the maximum value and its location which corresponds to the starting position for the bounding box along the horizontal axis. An example of the maximum value is shown in Figure 3(b).

**FIGURE 4.**
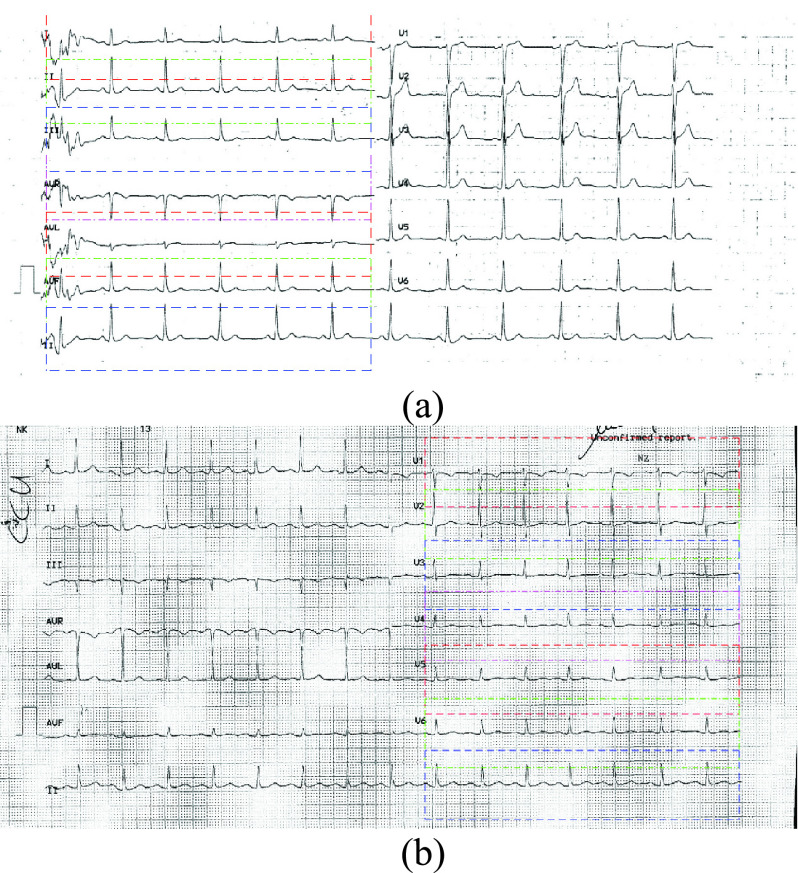
(a) Input ECG image with the obtained bounding boxes for the left part, (b) input ECG image with the obtained bounding boxes for the right part.

We then determined the different bounding boxes of the left half of the scanned ECG image as shown in Figure 4(a). The same approach was carried out in order to determine the bounding boxes of the right half of the scanned image by flipping the image along the vertical axis with an example output shown in Figure 4(b).

### C. Stage III. Extracting the ECG Signal

**FIGURE 5.**
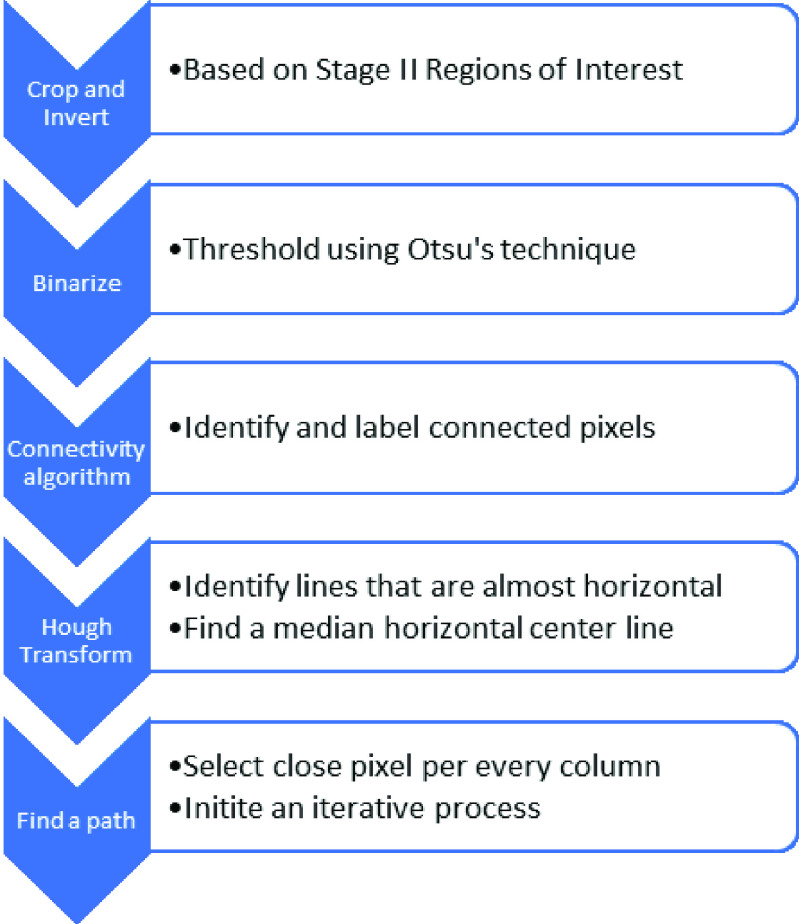
Signal extraction steps.

In a third stage, an image processing algorithm was implemented to identify the ECG signal in each bounding rectangle. The signal was extracted from the regions of interest or the bounding boxes detected in stage II. The extraction process is outlined with the main steps in Figure 5.

**FIGURE 6.**
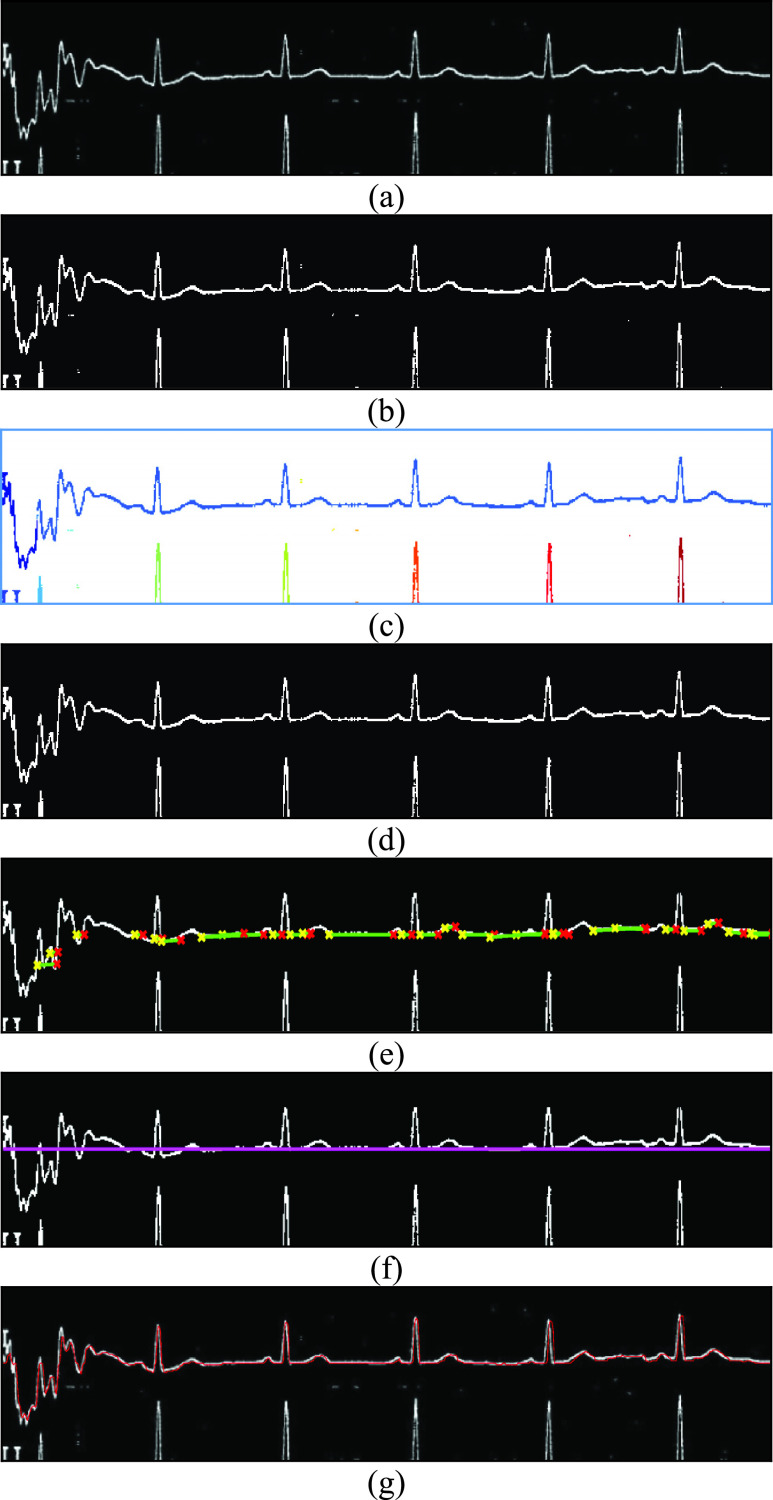
(a) Example of a cropped ECG segment with inverted colors, (b) corresponding binarized ECG segment, (c) corresponding labeled ECG image with different objects, (d) corresponding binarized ECG image with the smaller objects removed, (e) corresponding binarized ECG image showing horizontal lines detected with Hough Transform, (f) corresponding binarized ECG image showing the centerline of the ECG signal, (g) corresponding binarized ECG image showing the detected ECG signal in red.

After locating the different bounding boxes for each of the ECG signals, we applied the extraction process on each region and determined the ECG signal. As a first step, each bounding box was cropped and color-inverted giving a signal that is closer to a white color as shown in Figure 6 (a). Binary thresholding was next applied relying on Otsu’s automatic thresholding technique [11] to yield an intermediate image as shown in Figure 6(b). We multiply the automatic threshold by 1.2 to obtain the correct threshold to achieve binarization noting that this coefficient was achieved by testing for multiple values. Using connectivity algorithms [12], [13], we identified and labeled the different objects in the image as shown in Figure 6(c) where different labels are presented in different colors. The smaller objects were considered as noise and removed as shown in Figure 6(d).

Given that each ECG signal has an area where the signal moves linearly in a rather horizontal fashion, line detection was next applied using the Hough Transform [14]. This enabled identifying the lines that are almost horizontal as shown in Figure 6(e). The median value of all the lines was next calculated in order to determine the main centerline of the ECG signal as shown in Figure 6(f).

**FIGURE 7.**
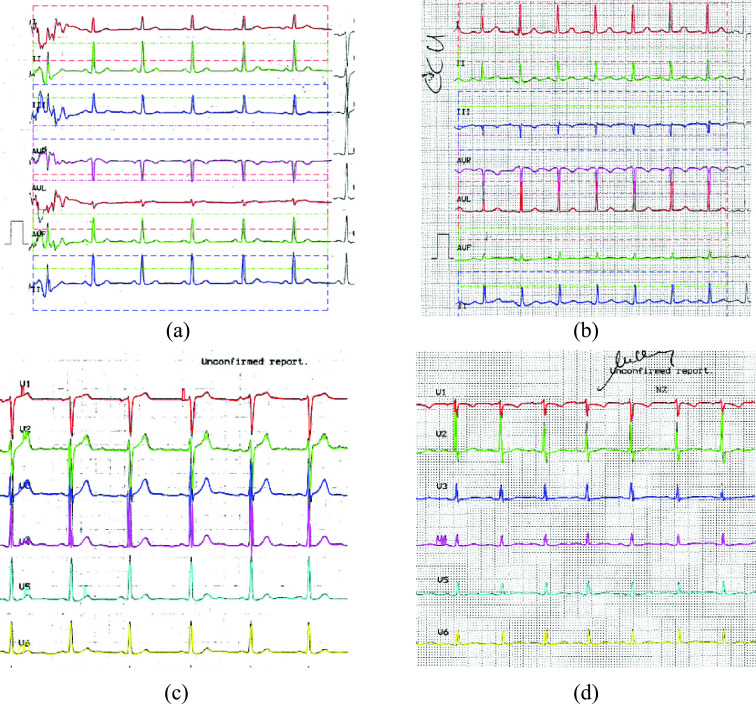
(a) Example of a printed ECG record showing the detected ECG signals of the left half, (b) another example of a printed ECG record showing the detected ECG signals of the left half (b) example of a printed ECG record showing the detected ECG signals of the right half, (b) another example of a printed ECG record showing the detected ECG signals of the right half.

The next step allowed to find a path from the left to the right of the image that was closest to the center-line by selecting one pixel per column. Initially, we selected all points close to the centerline. Next, we iteratively included points that are connected and adjacent to the already selected points. This step was repeated iteratively for a maximum of 20 times or until no more pixels can be checked for validity. The number 20 here is heuristic and was found by testing for several values as is the case for the different parameters used. Afterwards, and along each empty column, we selected the pixel that is farthest from the centerline. Finally, linear interpolation was used to calculate values for the columns that remain empty. An example of the obtained path is shown highlighted in red in Figure 6(g). Those steps were performed for all the regions of interest to detect the ECG signals captured from the different leads as shown in Figures 7(a) and 7(b) corresponding to the left halves of the input images in Figures 1(a) and 1(b) respectively. Also, Figure 7(c) and 7(d) show the obtained right halves.

Each obtained digitized signal was next smoothed using standard averaging with a span of 5 values, while excluding all the values that exceed 5 times the median value to maintain the peak information; this was considered since peaks are critical for ECG signals. The number 5 here was determined by testing for multiple values to provide suitable smoothing.

**FIGURE 8.**
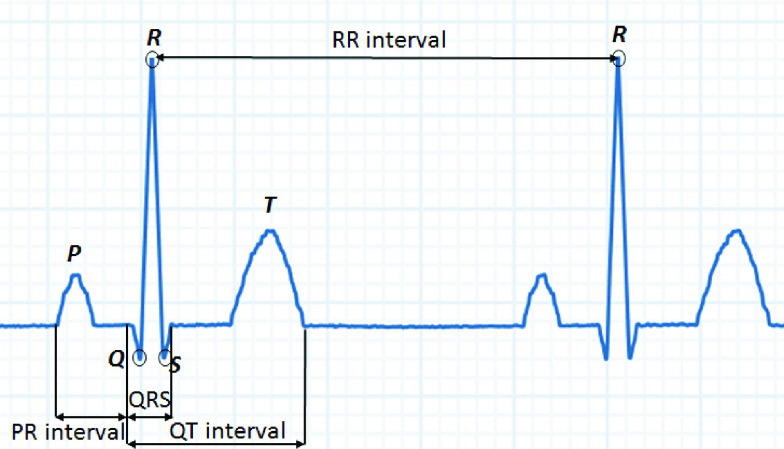
Examples of the PQRST pulse.

In addition, the PQRST points and intervals PR, QRS, RR and QT for a single beat were determined and calculated. The QRS interval was first detected using the Pan Tompkins algorithm [15]. P and T were next detected using peak information by utilizing the concept of PQRST pulse shown in Figure 8, since P is the nearest closest peak before Q, and T is the nearest highest peak after S. This calculation may not always be exactly accurate or indicative of the real PQRST values due to the utilization of the Pan Tompkins algorithm which might have some errors, but the same calculation is performed to both digitized and original signals.

**FIGURE 9.**
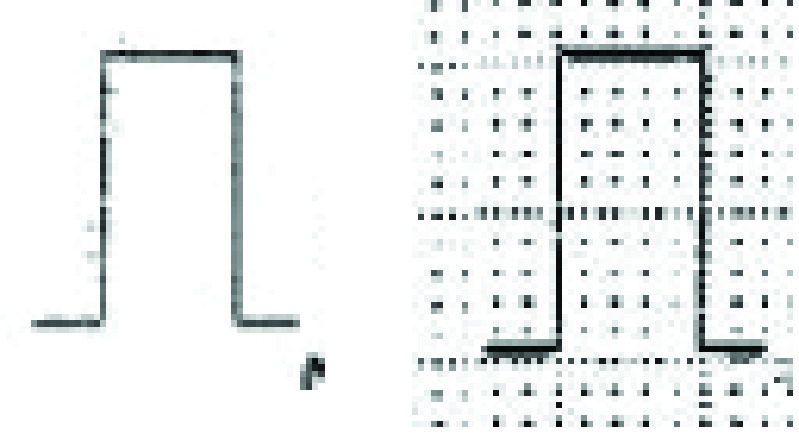
Examples of the reference pulse used to determine the scale of the extracted signals.

Finally, the scale of the ECG was extrapolated into the translational digitization method. Examples of the reference pulse are shown in Figure 9 with x-axis signals corresponding to milliseconds (ms) and y-axis signals to millivolts (mV). For this objective, the tool offered the possibility to crop the reference pulse to calculate the exact values of millivolts and milliseconds per pixel. Besides, the position of the scale indicator in the main ECG records that we targeted was always to the left of the AVF lead and below the AVL lead. This means that it is possible to directly detect the scale by considering a rectangle whose height equals 60% of the height of the region of interest described in section II and whose width is 75% of the rectangle’s height, and therefore detecting the scale indicator.

## III. Results

**FIGURE 10.**
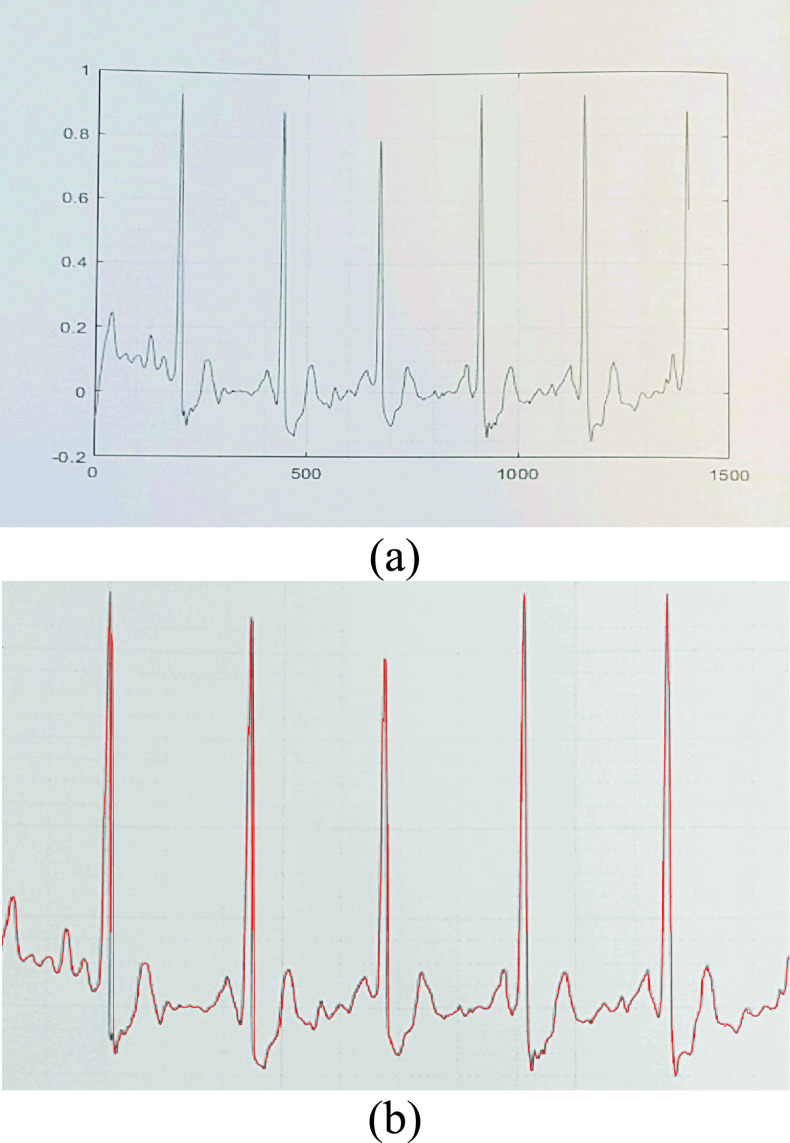
(a) Example of a scanned image that can be digitized, (b) corresponding scanned areas and the digitized curves.

The proposed method was validated using a database of thirty ECG signals extracted from a publicly available online dataset through Physionet [16]. These are used because we can use ECG in paper-based format and in digital format as well. All the images were of a jpeg format and each ECG curve were lying in an almost square image of a 1000$\times$1200 resolution. The scale information of the ECG record was taken into consideration to accurately calculate the extracted signal. The validation of the results was performed using varying signals from different records with each signal having a 1000 data-points interval and constituting of a minimum of 4 heartbeats. The signals were printed and afterwards scanned. An example of a scanned image is shown in Figure 10(a).

Afterwards, the proposed algorithm is applied to a selected part of the image and the corresponding area is digitized. Figure 10(b) presents an example of the obtained results with the digitized curves shown on top of the selected areas.

**FIGURE 11.**
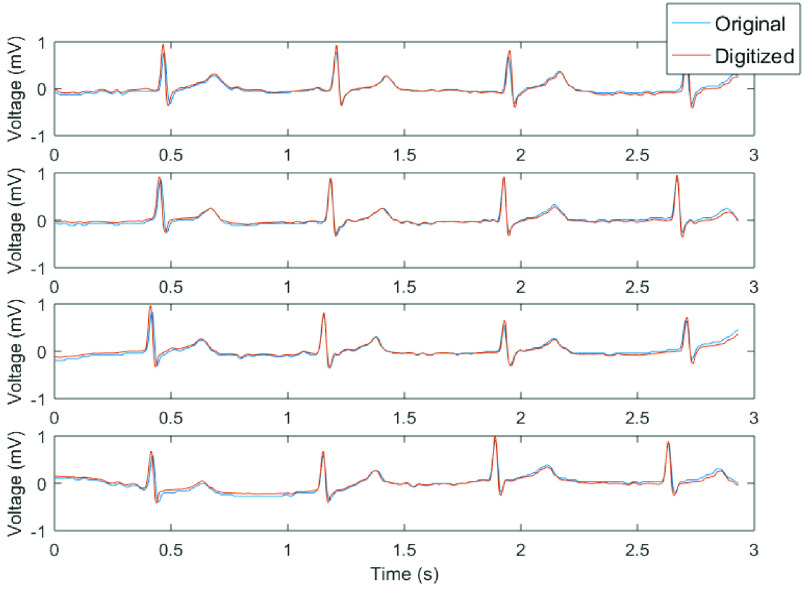
Plot showing paper ECG signal (blue) and digitized ECG signal (red) for four leads.

In addition, Figure 11 shows the tracings obtained from several of the retrospectively obtained digitized ECGs printed from the online database and compared to the original data itself. To measure the accuracy of the method, Pearson’s linear vector correlation coefficient was computed between the original ECG signal and the digitized ECG signal [17]. This coefficient is defined in equation 1, where *sig1* and *sig2* are the signals or vectors to be compared, *cov* is the covariance and $\sigma $ is the standard deviation. This measurement was used in [5] and others to indicate the accuracy which led to its choice.

\begin{equation*} \rho _{sig1,sig2} =\frac {\textrm
            {cov}(sig1,sig2)}{\sigma _{sig1} \sigma _{sig2}}\tag{1}\end{equation*}

Both signals were resampled to the same rate of 1000 data-points for each; this was done to adjust to the greater sampling rate of the original signal. In addition, the sampling rate of the digitized ECG signal varied according to the resolution of the scanned image. Down-sampling was not performed as it has proven to decrease the correlation between both signals.

The correlation values between the original signals of the thirty ECGs and their corresponding digitized signals were found to be between 0.85 and 0.99 with a mean value of 0.952 and a standard deviation (0.036). In addition, The PQRST points and intervals PR, QRS, RR and QT for a single beat were also validated in comparison to the original ECGs. The correlation values for all the intervals matched significantly the online digital database with correlation values 0.981-1. Table 1 presents the different results for the correlation values in addition to the computed p-values for both the time and voltage axis. It is important to note that the different parameters can be deduced from the PQRST pulse of Figure 8 where the x-axis indicates the time (ms) and the y-axis indicates the voltage (mV).

**TABLE 1:** Matching Results Between Original and Digitized ECGs

Parameter Name	Correlation Value	Standard Deviation	P-value	95% Confidence Interval	
				LCL	UCL
Overall Matching (mV)	0.952	0.036	< 0.001	0.9391	0.9649
PR interval (ms)	0.984	0.021	< 0.001	0.9802	0.9878
QRS interval (ms)	1	<0.001	< 0.001	1	1
QT interval (ms)	0.981	0.023	< 0.001	0.9769	0.9851
RR interval (ms)	1	<0.001	< 0.001	1	1
R values (mV)	0.838	0.082	<0.001	0.8087	0.8673
P values (mV)	0.833	0.079	<0.001	0.8047	0.8613
Q values (mV)	0.927	0.024	<0.001	0.9184	0.9356
S values (mV)	0.822	0.0676	<0.001	0.7978	0.8462
T values (mV)	0.781	0.077	<0.001	0.7534	0.8086

In order to provide a comparison with previous work, Table 2 depicts the results of this work and the work in [5] noting that they relied on observed values and utilized totally different data, but we used actual digital data with exact values. This comparison is not exact due to the varying data but is depicted here to provide insight into the obtained results.

**TABLE 2:** Main Matching Results Comparison With the Work in [5]

Parameter	Obtained Value	Value in [5]
Sample Size	30 ECGs	10 ECGs
PR Correlation	0.984	0.985
QRS Correlation	1	0.803
QT Correlation	0.981	0.902
RR Correlation	1	0.993

It is worth noting that the RR accuracy indicates that of the heart rate which means that the proposed approach achieves 100% heart rate precision.

## IV. Discussion

We aimed in this study to describe a high quality tool for ECG digitization that presents several advantages over previous methodologies. An image processing method was implemented to detect the ECG regions of interest and extract the ECG signal. This was followed by serial steps that digitize and extract vector data from the results. The model was compared with an online database of digitized ECGs and evaluated by correlation statistics to assess fidelity and accuracy of the proposed model. The method was shown to be significantly matched to the digital database of ECGs (correlation value 0.952) with good correlation with the ECG intervals (correlation value 0.981-1).

Other methods in the literature reported use of a Matlab-based solution and Laplacian image filtering [18], de-skewing operation using Hough transformation [19], and others [20], [21]. In addition, the work in [10] aimed to extract samples as text from ECG strips to provide ECG analysis. The authors of [6] utilized a relatively simple approach to work on a single ECG curve using colored images while mainly relying on thresholding. Moreover, the work in [8] targeted 12 lead ECG digitization while handling each lead on its own. Their proposed image processing algorithm mainly relies on de-skewing the scanned ECG using Radon Transform followed by adaptive binarization in addition to morphological operations. Furthermore, [7] presented an entropy-based bit plane slicing for ECG digitization while targeting colored records. In addition, the authors in [22] targeted ECG digitization for a recorded database with 7203 participants. Their work utilized the ECG Trace Tool for digitization which is not publicly available. The work in [22] concentrated on user interaction and feedback.

Besides, the work in [23] provided a review and a summary of previous works addressing digitizing paper electrocardiograms noting the status and the challenges such as noise, alignment, and grid issues. The authors of [23] emphasized on the importance of ECG digitization due to the useful information extracted from the ECG record. They further noted that [9] provided a solution for several issues. The work in [9] can be considered as one of the pioneering works that addressed ECG scanning. Authors of [9] presented an image processing engine that can be initially used to detect the grid of the ECG document followed by digitizing the ECG waveforms using active contour modeling. This is the only previous work in literature that addressed detecting the curves prior to detecting the waveforms, but this approach relied on having a grid with known parameters such as distance between grid lines, paper speed and others which are not always available and require user involvement. The research in [9] also highlighted the limitations of the proposed approach such as noise level and using black and white photocopies which may lack the grid points. Our work solved these issues in a robust manner as explained throughout this paper.

In addition, and since this work provides an automated approach to detecting the location of each lead, this method may not be applicable to all kinds of records such as those where three leads are along the same row as opposed to two which is the case in Figures 1(a) and 1(b). However, the proposed approach can be generalized to work with different cases where there is a varying number of leads such as one or three leads along the same image or where less than six leads are in each half of the ECG scanned image.

It is important to emphasize that this step of digitization can be followed by the analysis of the extracted data to yield the ECG interpretation which is the main objective of our future work to aid in patient diagnosis. The application of such approaches has already been interrogated and are currently advancing rapidly in the rising field of wearable devices [24], [25]. These are expected to find wider application into the hospital setting with further amplification in the precision and accuracy of a desired outcome through machine learning algorithms that integrate data from echocardiography, cardiac nuclear imaging, cardiac MRI, or angiography [26].

Moreover, it is important to note that removing the proposed smoothing discussed in section III leads to a slightly reduced accuracy of the overall matching with a mean correlation value of 0.948 and degrades the mV correlation values by more than 3% for each of the peaks.

Besides, basic testing of the available Android Application [27] has proven that the proposed approach provides better accuracy since the application often failed to detect the curve from the start which made comparing based on correlation values irrelevant. Moreover, the paper provides the millivolt correlation accuracy which was not provided in the previous work in [5] and the results here surpass those of [5] noting that the work in [5] utilized OCR to acquire patient data which was not used here in addition to batch processing. We do acknowledge that the tool does not tackle bulk/mass processing at a time which is intricately tied to the interface that depends on the user input. This process, even though slower in bulk data, allows for a more accurate ECG reading tool.

**FIGURE 12.**
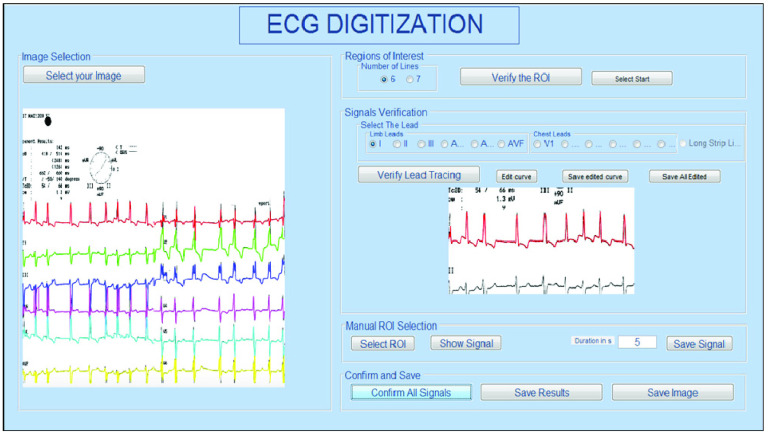
Layout of the digitization tool.

The power of the method used here refers to an automated crop feature that extracted different ECG regions of interest. The method is implemented as a computer tool having an interface that allows a simple and fast digitization process. The graphical user interface offers the capability to select the number of leads, verify the regions of interest, specify and visualize each detected lead signals individually, perform the full digitization, and save the resulted signals in Excel and image forms. In addition, a scaling sub-menu is offered to calculate the exact values of the conversion between pixels and voltage or time. To visualize the key characteristics of the ECG, a cursor mode is also offered so that the user can track the curves points showing the time and voltage at each point. Moreover, the user can insert an additional cursor showing the difference in time and voltage relatively to the first cursor. Such interface can help cardiologists in the diagnosis and the storage of the digitized signals. A sample screenshot displayed by this tool is shown in Figure 12, and a video summarizing its main functionalities is available at: https://www.youtube.com/watch?v=r7uKny4z8gw

Besides this, the software provides the user with the capability of editing the found curve by dragging the points to the suitable location, which means the accuracy of the selected curve can be improved by the user.

## V. Conclusion

This study presents a novel method that utilizes standard image processing techniques for the digitization of ECG signals from previously stored paper or scanned format. The approach reconstructs ECGs with an average of more than 95% correlation matching. This method was embedded into a user-friendly software tool that offers a simple interface tailored for cardiologists and researchers. This can permit integrating the digitized ECG signal of rare and old medical cases with other cardiac data in machine learning algorithms to aid in the diagnostic and prognostic evaluation of patients with cardiovascular disease.
